# Loss of miR-198 and -206 during primary tumor progression enables metastatic dissemination in human osteosarcoma

**DOI:** 10.18632/oncotarget.26284

**Published:** 2018-11-06

**Authors:** Steven Georges, Lidia Rodriguez Calleja, Camille Jacques, Melanie Lavaud, Brice Moukengue, Fernando Lecanda, Thibaut Quillard, Marta Tellez Gabriel, Pierre-François Cartron, Marc Baud’huin, François Lamoureux, Dominique Heymann, Benjamin Ory

**Affiliations:** ^1^ INSERM, UMR-S 1238, Nantes 44035, France; ^2^ PhyOs, Sarcomes Osseux et Remodelage des Tissus Calcifiés, Université de Nantes, Nantes 44035, France; ^3^ Division of Oncology, Adhesion and Metastasis Laboratory, Center for Applied Medic al Research, University of Navarra, Pamplona, Navarra 31008, Spain; ^4^ Equipe Apoptose et Progression Tumorale, Centre de Recherche en Cancérologie et Immunologie Nantes Angers, CRCINA, INSERM, U1232, Université de Nantes, Université d'Angers, Nantes 44035, France; ^5^ LaBCT, Institut de Cancérologie de l'Ouest, Saint Herblain 44800, France; ^6^ European Associated Laboratory Sarcoma Research Unit, INSERM, University of Sheffield, Sheffield S10 2TN, UK; ^7^ Cancéropole Grand-Ouest, Réseau Epigénétique (RepiCGO), France

**Keywords:** osteosarcoma, microRNAs, metastasis, C-Met

## Abstract

The metastatic dissemination is a complex multistep process by which tumor cells from a primary site enter into the systemic circulation to finally spread at distant sites. Even if this mechanism is rare at the tumor level, it remains the major cause of Osteosarcoma-patients’ relapse and mortality. MicroRNAs (miRNAs) have recently been described as novel epigenetics’ genes’ expression regulators actively implicated in cancer progression and dissemination. The understanding of their implication in the metastatic spreading could help clinicians to improve the outcome of osteosarcoma. We established the miRNA’s expression-profile between primary bone-tumors (PTs), circulating tumor cells (CTCs) and lung metastatic (META) samples from *in vivo* mice xenograft models. Our results show that the expression level of the miR-198 and -206 was decreased in META samples, in which the expression of the metastasis-related receptor C-Met was up-regulated. Those expression variations were validated in osteosarcoma patient biopsies from matching primary tumors and lung metastasis. We validated *in vitro* the endogenous miRNAs inhibitory effects on both migration and invasion, as well as we confirmed by luciferase assays that the C-Met receptor is one of their *bona-fide* targets. The anti-metastatic effect of these miRNAs was also validated *in vivo*, as their direct injections into the tumors reduce the number of lung-metastases and prolongs the overall survival of the treated animals. All together, our results suggest the absence of the miR-198 and -206 as powerful predictive biomarkers of the tumor cell dissemination and the rationale of their potential therapeutic use in the treatment of Osteosarcoma.

## INTRODUCTION

With an incidence of 4 to 5 cases per million diagnosed in the United-States, Osteosarcoma is the most common primary malignant bone-tumor [1, 2]. It mainly affects children and young adults and is characterized by an osteoid neo-formation associated with osteolysis, principally occurring at the metaphysis of the long bones [3]. The current treatments of this disease are usually based on a combination of surgery and chemotherapy that have markedly improved the five-year survival rate to 60–70%. However, a significant proportion of the patients still responds poorly to chemotherapy regiments and has a high risk of local relapse or distant metastasis spreading, even after surgical resection [4, 5]. The achievement of the drug’s toxicity thresholds and the presence of lung metastasis at the diagnosis time-point are the two major hindrances of the proper Osteosarcoma’s cure. Despite all the therapeutic-optimization’s efforts, the metastatic dissemination still dramatically compromises the cancer patients’ survival and a better understanding of its underlaying mechanisms is needed.

Several studies support the implication of the well known Hepatocyte Growth Factor (HGF) Receptor, C-Met in the regulation of the metastatic dissemination in a wide variety of malignancies including hepatocellular carcinoma [6], lung [7], breast [8] and colon cancers [9], as well as Osteosarcoma [10]. Understanding the mechanisms which modulate the expression of this receptor is thus of particular importance in this context. The epigenetic regulations mechanisms involving the microRNAs are just emerging and still have to be deciphered.

MicroRNAs (miRNAs) are small, 18- to 24-nucleotides non-coding RNAs that regulate gene expression by targeting the 3′ untranslated region (3′UTR) of messenger RNAs (mRNAs), resulting in either mRNA cleavage or protein-translation’s repression [11, 12]. Since the discovery of the first development-related miRNA in *C. elegans* [13], hundreds of others have been identified in many species, including *Homo sapiens* [14]. These epigenetic regulators are involved in plethora of natural biological processes such as proliferation, differentiation, development or apoptosis, but they have also been found to play a major role in tumorigenesis [15, 16]. Indeed, as their expression is often altered in cancer, their deregulation is furthermore frequently associated with the pathological stage of the disease. For instance, it was reported that such deregulation affects the Osteosarcoma progression, chemoresistance and metastatic dissemination [5]. The miR-183 was indeed found to be down-regulated in Osteosarcoma and its expression level was correlated with the one of the Ezrin, a protein that affects motility and invasion and which also confers the required survival advantages allowing the cells to reach the lungs [17]. In addition, it was demonstrated that restoring the miR-143′s expression in Osteosarcoma cells has functional effects both *in vitro* and *in vivo*, as it reduces the cell viability, induces apoptosis and suppresses the lung metastases [18]. Such effects were mediated through its ability to target the anti-apoptotic factor BCL-2. Finally, the miR-26a and -454 were also down-regulated in Osteosarcoma and as the expression of the former correlates with the poor prognosis of the patients and their metastatic occurrence, the latter displays anti-proliferative and anti-invasive features through its direct targeting of C-Met [19, 20]. All those evidences sustain the fact that the miRNAs are promising tools in an attempt to better understand the processes that drive malignancies, from their onset to their metastatic spreading. In line with these considerations, the miRNA-mediated control of the C-Met receptor’s expression is of particular interest in the context of the Osteosarcomas’ metastatic dissemination.

This was thus the purpose of this study, whose departing point was the differential expression analyze of the miRNA from primary bone tumors (PTs), Circulating Tumor Cells (CTCs) and lung metastases (MET) from an *in vivo* xenograft model of Osteosarcoma. We identified both the miR-198 and the miR-206 as two miRNAs only expressed in PTs. We have shown that their loss by some tumor cells permit them to acquire migrative and invasive capabilities, allowing them to detach from primary tumor sites, enter into the systemic circulation and grow at distant sites. By artificially modulating their expression in Osteosarcoma cells and by performing *in vitro* luciferase reporter assays, we confirmed that the Hepatocyte Growth Factor Receptor C-Met was a *bona-fide* target of these miRNAs. Such results consequently corroborate the fact that an increased expression of this receptor was found in metastases samples from both our *in vivo* model and from Osteosarcoma patients.

In a clinical approach, our work thus adds a novel glimpse at the possibility to use the miR-198 and -206 as novel molecular prognosis markers of the Osteosarcoma’s metastatic spreading. In addition, this study shed lights on the potentiality to avoid the poor outcome of Osteosarcoma through restoring a sufficient expression level of these miRNAs into the tumors, which could be a hopeful therapeutic strategy for the future.

## RESULTS

### A set of miRNAs differentially expressed in primary tumors (PTs), circulating tumor cells (CTCs) and metastatic samples (METs) potentially targets the C-Met receptor for inhibition

**Figure 1:**
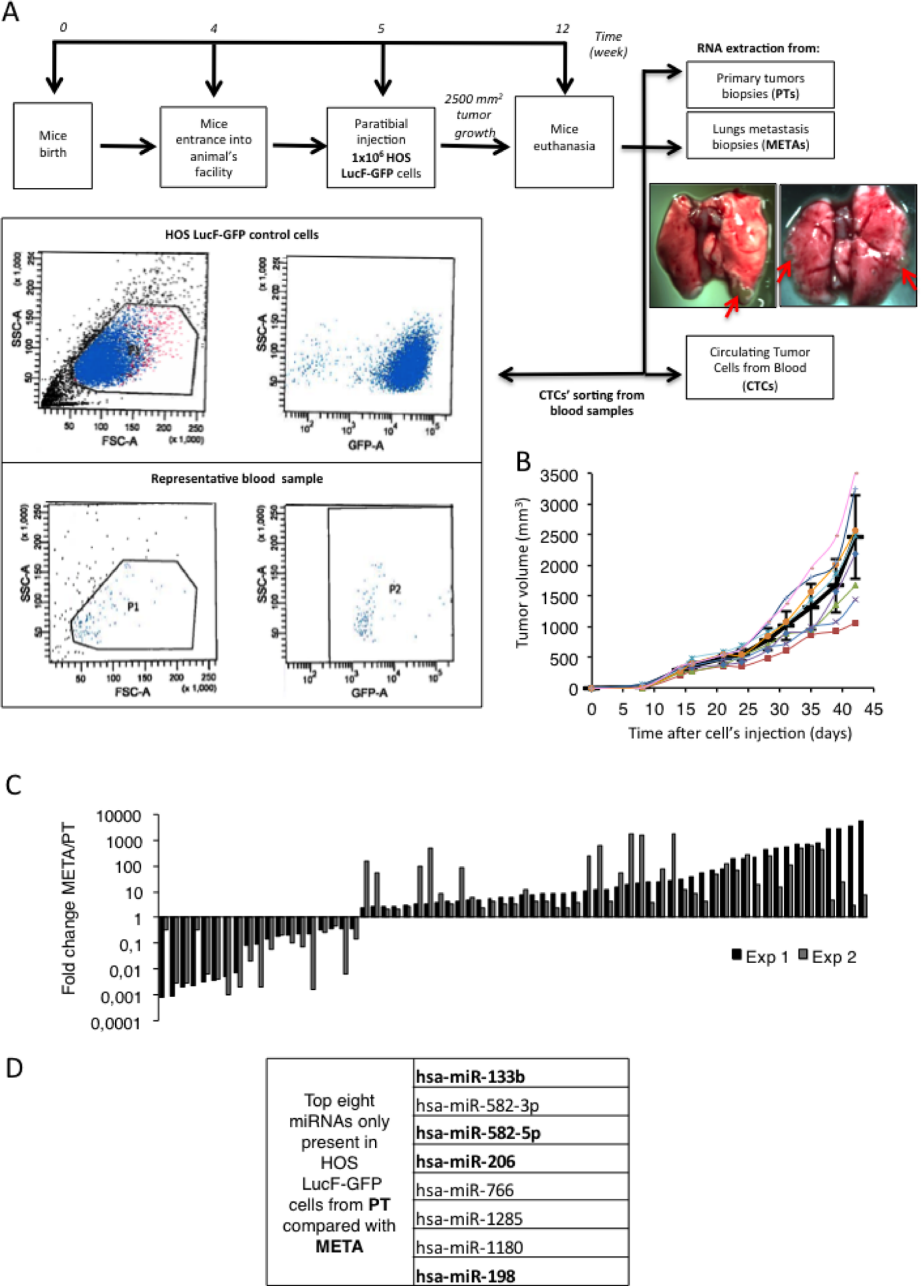
A set of miRNAs differentially expressed in primary tumors (PTs), circulating tumor cells (CTCs) and metastatic samples (METs) potentially targets the C-Met receptor for inhibition (**A**) Experimental design: 1 million HOS LucF-GFP Osteosarcoma cells paratibially injected in nude mice. The mice were sacrificed when the tumor volume reach 2500 mm^3^ and samples from Primary bone tumors (PTs), Circulating Tumor Cells (CTCs) and metastatic nodules (METs) were collected and subjected to RNA extraction (upper panel). The lower panel shows the two scatter plots used to isolate the CTCs (representative of the 2 experiments performed). cell-granulometry (SSC) in function of the cell-size (FSC) (left panel) and SSC in function of the GFP-fluorescent signal (right panel). Both top scatter plots illustrate the control conditions used as a reference for the blood-sample CTCs’ isolation, composed of the HOS LucF-GFP cells cultured *in vitro*. Both bottom scatter plots illustrate the results from the CTCs sorted from the blood-samples and P2 is composed of the CTC population recovered in one of the experiments. (**B**) Tumor volumes (mm^3^) of eight mice paratibially injected with HOS LucF-GFP cells. Average tumor volume is represented by the black thick curve. Tumor volumes were calculated with the formula (t^2^ × l)/2 where t is the tumor thickness and l is the tumor length. Error bars show s.e.m. (**C**) Differential miRNAs’ expression-profiles assessed by Taqman low density array analysis. Each histogram shows both the fold change and the direction of change for one given miRNA’s expression regulated more than 2-folds between the PTs and the METs samples from two independent experiments (Exp 1 and Exp 2). RNU6B, RNU44 and RNU48 were used as housekeeping genes and the miRNA’s expression from the METs is normalized on the PT one. (**D**) List of the eight microRNAs over the 760 analyzed, found to be expressed only in PTs. Analysis performed using TargetScan, DianaLab and miRANDA databases’ algorithms. Four miRNAs were found to potentially target the C-Met receptor (bolded miRNAs).

In order to better understand to what extent the miRNAs could be involved in the metastatic spreading of the Osteosarcoma, we analyze the miRNA-profiles of bone PTs, CTCs and lung META samples obtained from an *in vivo* orthotopic xenograft model of Osteosarcoma. 1.5 million of human Osteosarcoma HOS LucF-GFP cells were thus paratibially injected in athymic mice (Figure 1A, upper panel). The tumor growth was assessed and the animals were sacrificed when the tumor’s volumes reached 2500 mm^3^ (Figure 1B). At the time of euthanasia, samples of both the bone PTs and METAs were collected from the lungs of the animals, as they are the preferentially site of metastastatic dissemination in this model. CTCs were isolated from the systemic blood by cell sorting facilities, based on the granulometry, the size and the GFP-fluorescence properties of the injected tumor cells. An average of three hundred CTCs were isolated in each experiment (Figure 1A, bottom panel).

The total RNA of each kind of sample was extracted and the expression-profile of 760 known-miRNAs was established by performing Taqman low density arrays (TLDAs). The miRNA’s expression-profiles obtained in the METAs samples were normalized on the ones of the PTs samples in two independent experiments (Figure 1C). To go further in our study, the strategy chosen was to focus only on the miRNAs found in PTs whose the expression is lost during the course of the metastatic spreading already at the stage of CTCs. It suggests that the target-genes normally repressed by these anti-metastatic miRNAs are involved in pathways of paramount importance in promoting the migration of the cancer cells. Eight miRNAs were thus identified as being the most under-expressed in the CTCs and the metastatic cells compared to the PTs (Figure 1D).

These candidate miRNAs were then subjected to an *in silico* analyses using the algorithms provided by TargetScan, DianaLab and miRANDA databases, in order to identify common putative targets involved in the metastatic dissemination. These analyses reveal that four of them; namely the miR-133b, -198, -206 and -582-5p were predicted to target the C-Met receptor, a well-known protein that triggers the metastatic C-Met pathway (Figure 1D, bolded miRNAs).

### Validation of candidate miRNAs repression and C-Met overexpression in the lung-metastasis (METs) samples from both mice and patients’ samples

**Figure 2:**
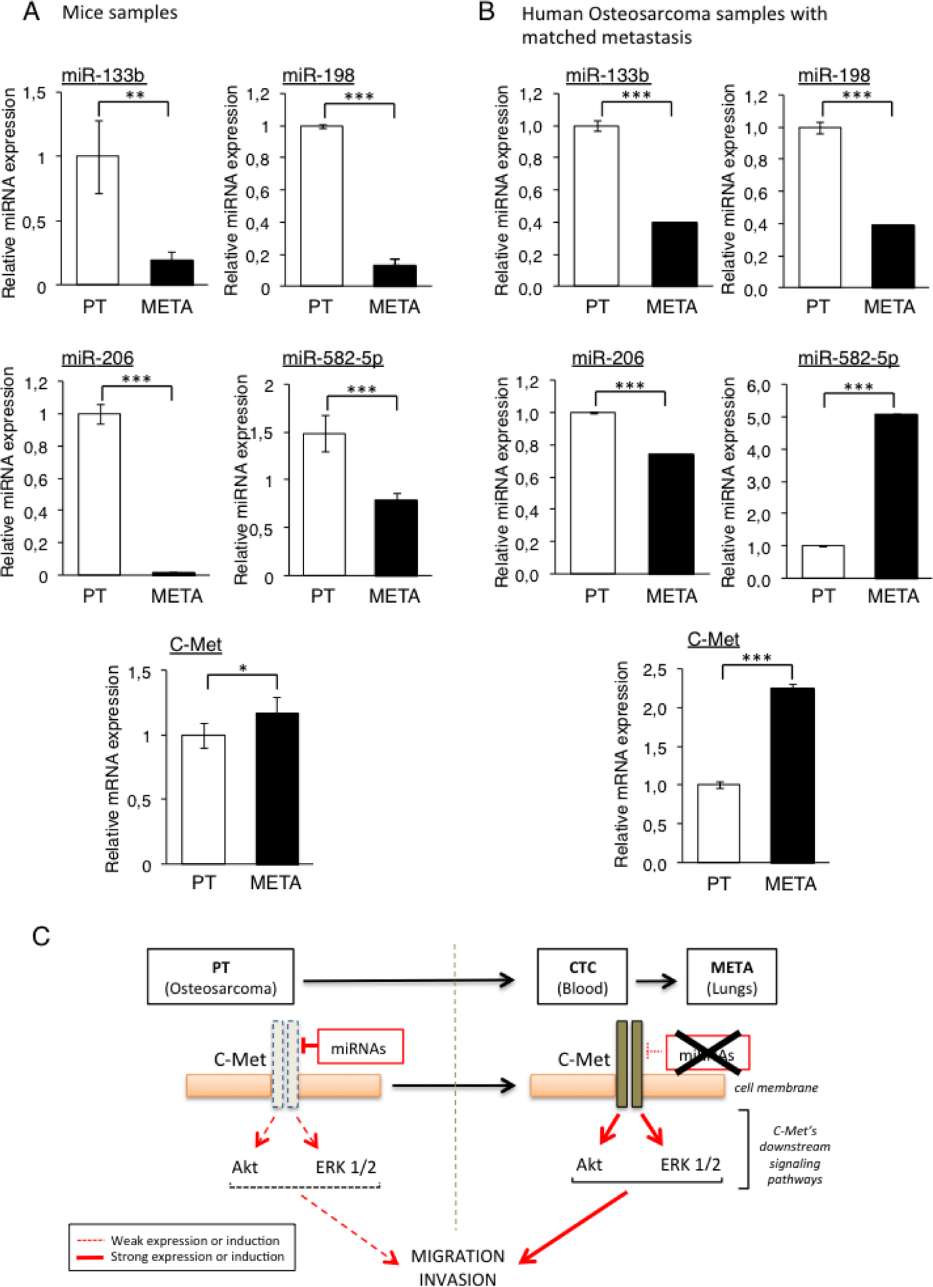
Validation of candidate miRNAs repression and C-Met overexpression in the lung-metastasis (METAs) samples from both mice and patients’ samples The four candidate miRNAs’ expression (miR-133b, -198, -206 and -582-5p) as well as C-Met were analysed by RT-qPCR from xenografted-Osteosarcoma from mice samples (**A**) and from four metastatic Osteosarcoma patients (**B**). The expression levels were compared between primary tumors (PTs) and their associated metastases (METs). ^*^p < 0.05, ^**^p < 0.01, ^***^p < 0.001. One-way ANOVA and Dunnett’s multiple comparison tests were used to compare the significance of the results. Average expression and error bars (s.d.) are represented for *n =* 3 measurements from representative experiments. RNU6B, GAPDH and B2M are used as housekeeping genes. (**C**) Rationale of the hypothetic mechanism: cells from the primary bone tumor (PT) express the candidates miRNAs predicted to target C-Met, thus maintaining a low activity of this receptor and its downstream signaling pathways, keeping the PT cells at their initial site. The miRNAs’ loss of expression in Circulating Tumor Cells (CTCs) and metastatic nodules (METAs) releases the pressure exerted on C-Met, resulting in increasing both its expression and activity, thus enhancing the metastatic potential of those cells.

To confirm that the four previously selected miRNAs, namely the miR-133b, -198, -206 and -582-5p are indeed under-expressed in the METAs samples compared with the PTs ones, we analysed their expression by RT-qPCR in each PT sample and the corresponding META from our initial *in vivo* model. We could indeed validate the TLDA’s results, as the miR-133b and -198 were found to be overexpressed 5-fold and 8-fold respectively, in PTs compared with METAs (Figure 2A). In addition, the expression of the miR-206 is almost ablated in METAs, validating the previous Taqman results. The miR-582-5p also follows the same expression-profile with however a less pronounced variation, less than 2-fold.

Because all the candidate miRNAs are predicted to target the C-Met receptor or are already validated as *bona-fide* C-Met inhibitor, the mRNA level of this gene was also assessed in the same samples. Interestingly, and inversely to the candidate miRNAs, we found that the C-Met receptor is more expressed in METAs than in PTs samples (Figure 2A).

In order to confirm and to give more accuracy to our *in vivo* results, we performed the same analyze with samples from four metastatic Osteosarcoma patients. The expression of the four candidate miRNAs was thus analyzed by RT-qPCR in PTs and in their corresponding lung metastasis. As observed in the *in vivo* model, all the microRNAs except the miR-582-5p are more expressed in PTs than in METAs. The expression of the miR-133b was indeed decreased by 2.5-fold, the one of the miR-198 by 1.4-fold and the one of the miR-206 by 2.6-fold (Figure 2B). Consistent with the miRNAs’ expression, we found that their putative target c-Met was 2-fold more expressed in METAs than in PTs. Given the inconsistent results obtained with the miR-582-5p regarding our starting hypothesis, this miRNA was excluded from the further investigations.

All this data contributes to establish the rationale of the starting hypothesis suggesting that the loss of expression of the candidate miRNAs by some tumor cells located within the heterogeneous primary Osteosarcoma is a selecting factor for them in order to spread into the whole body (Figure 2C). As these miRNAs are potential or validated repressors of C-Met, their lost in such cells could result in an increased expression of this receptor, giving them a metastatic potential.

### Direct inhibition of the C-Met’s protein’s translation by miR-198 and -206 modulates the downstream Akt and the ERK ½ signaling pathways

**Figure 3:**
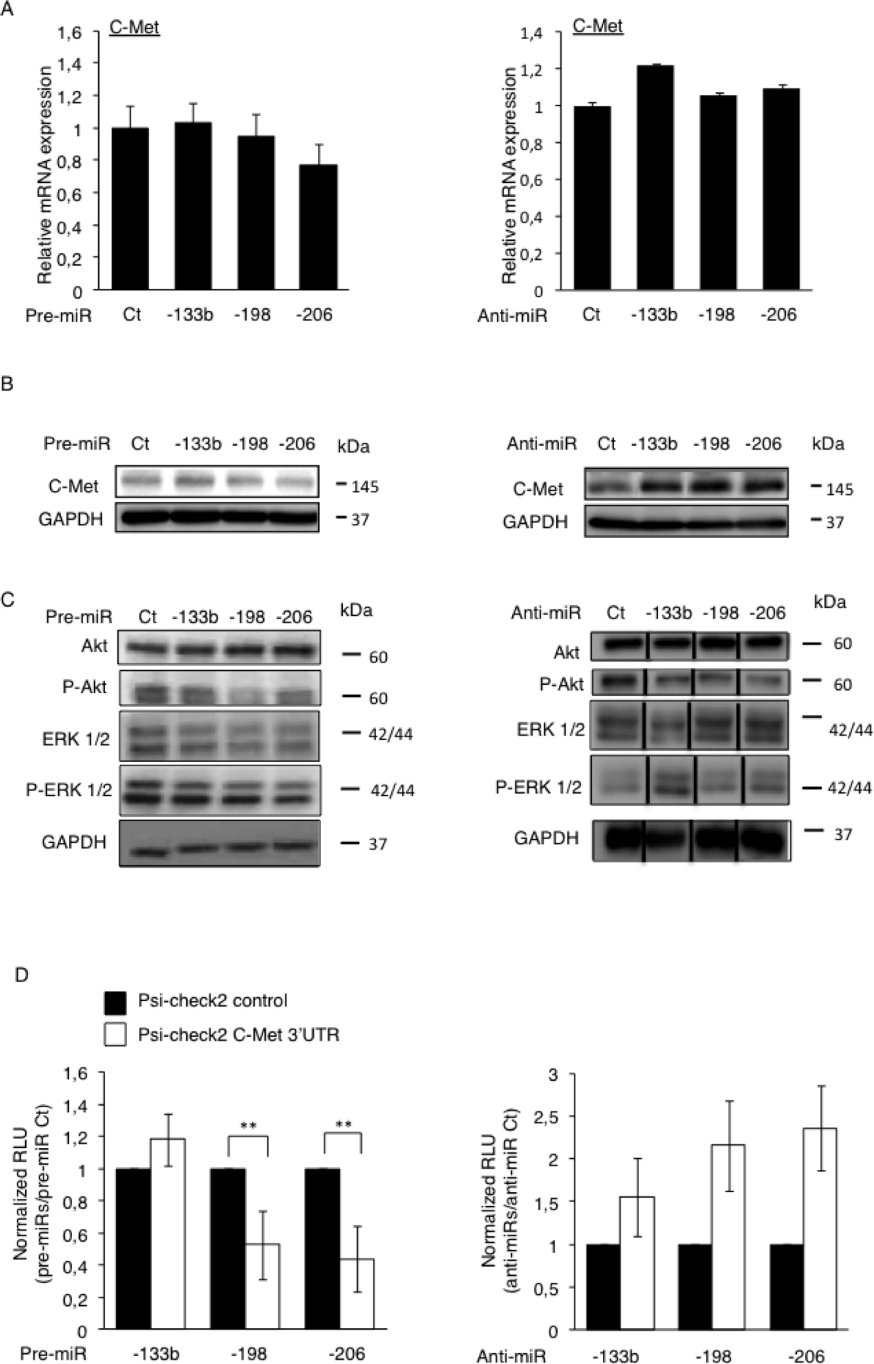
Direct inhibition of the C-Met’s protein’s translation by miR-198 and -206 modulates the downstream Akt and the ERK ½ signaling pathways The expression of C-Met was assessed at mRNA level by RT-qPCR (**A**) and at protein level by Western blotting (**B**) in the HOS LucF-GFP Osteosarcoma cell line pre-miRNAs’ (left panel) or anti-miRNAs’ transfections (right panel). Error bars show s.d. for *n =* 3 measurements from representative experiments. GAPDH and B2M were used as housekeeping genes. (**C**) The expression of Akt, P-Akt, ERK1/2 and P-ERK1/2 was assessed by Western blotting in the HOS LucF-GFP Osteosarcoma cell line 48 hours after either the pre-miRNAs’ transfections (left panel) or the anti-miRNAs’ ones (right panel). For all the Western blots presented, the GAPDH was used as an internal loading control. (**D**) The HOS LucF-GFP Osteosarcoma cells were co-transfected with the indicated pre-miRNAs (left panel) or anti-miRNAs (right panel), together with either the UTR-reporter (Psi-check2 C-Met 3′UTR, white bars) or the control vector (Psi-check2 control, black bars), the cells were lysed forty-eight hours after transfections and the luciferase bioluminescence was assessed. Results are shown as relative luciferase units (RLU) normalized to the control pre/anti-miR, and the control vector’s results were assigned to 1. ^*^p < 0.05, ^**^p < 0.01, ^***^p < 0.001. Error bars show s.d. for *n =* 3 measurements from representative experiments. One-way ANOVA and Dunnett’s multiple comparison tests were used to compare the significance of the results.

After confirming that the loss of expression of the miR-133b, -198 and -206, altogether with the concomitant up-regulation of C-Met was a feature of Osteosarcoma-metastases, we would like to confirm the C-Met receptor as a *bona-fide* target of these miRNAs. Through an *in vitro* approach, we artificially modulated the expression level of the three candidate miRNAs, by transfecting either the pre-miRNAs (miRNA mimics) or their corresponding anti-miRNAs (antisense oligonucleotides, complementary to the specific target-miRNA, inducing its degradation [21]) into HOS LucF-GFP Osteosarcoma cells. The expression of *C-Met* was assessed at mRNA level 72 hours after transfections and no significant differences were observed regarding the miRNAs-levels’ modulations (Figure 3A).

However, as miRNAs can regulate the gene’s expression by only preventing the protein-translation instead of directly inducing the degradation of the corresponding mRNA [22], the miRNA-mediated inhibition of C-Met has been investigated at the protein level. The C-Met protein’s expression was thus analyzed by Western blotting 48 hours after transfections of our three candidates pre-miRNAs or anti-miRNAs (Figure 3B). Although only a slight reduction of the C-Met’s expression was observed consequently to the pre-miRNAs transfections (Figure 3B, left panel), it is obvious that an increased expression of the receptor was obtained when the endogenous miRNAs are inhibited, especially in the anti-miR-198 and -206’s conditions (Figure 3B, right panel).

To functionally support the inhibitory-power of these miRNAs on the expression of C-Met, we assessed the activation’s status of the Akt and the ERK ½ signaling, two of the C-Met’s downstream pathways. We thus evaluated the phosphorylation level of Akt and ERK ½ by Western blotting, forty-eight hours after either the pre-miRNAs or the anti-miRNAs’ transfections (Figure 3C). Although they have no effect on the total Akt amount, all the miRNAs reduce the P-Akt level, especially the miR-198 (Figure 3C, left panel). Furthermore, they also inhibit the activation of the MAPK pathway, as illustrated by the reduced intensity of the 44 kDa-ERK1-corresponding bands. As expected, the anti-miRNAs, in particular the miR-133b and the -206 ones display the opposite effect on this pathway, by increasing the phosphorylation level of both ERK1 and 2 (Figure 3C, right panel). Surprisingly, and contrary to all expectations, the anti-miRs seem to decrease the phosphorylation of Akt. Nevertheless, this data even support that these miRNAs have functional consequences on the regulation of pathways which are normally activated by the C-Met receptor.

Finally, to further assess if the candidate miRNAs directly modulate the C-Met’s expression through a direct targeting, we performed a luciferase reporter assay, by transfecting a luciferase-bearing vector in which the 3′UTR of C-Met replaces the one of the luciferase gene. Such vectors were thus concomitantly transfected with each candidate pre- or anti-miRNA and the luciferase activity was measured forty-eight hours after transfections. A significant decrease in the bioluminescence was observed consequently to the pre-miRNA-198 and -206’s transfections, 37 and 45% respectively compared to the control conditions, meaning that both miRNAs are truly able to bind to the 3′UTR of C-Met (Figure 3D, left panel). Nonetheless, no similar effect was observed with the pre-miR-133b. As expected, an increase in the measured-bioluminescence was obtained in the case of the anti-miRNAs’ transfections compared with the control conditions, even if the changes monitored were not statistically significant (Figure 3D, right panel).

Curiously, even if Hu *et al.*, have previously demonstrated the direct targeting of C-Met by the miR-133b in a colorectal cancer model [23], the inconsistency in the results of both the luciferase-assay and the Western Blot analysis obtained with this miRNA does not allow us to get the same conclusion, thus we have chosen to exclude this miRNA from further studies. In agreement with the studies from Tan *et al.*, and from Yan *et al.*, our data give evidence that C-Met is a *bona-fide* target of both the miR-198 and -206, these ones exerting their epigenetic inhibitory functions through preventing the translation of this receptor into protein [24, 25].

### miR-198 and -206 inhibit both the migration and invasion capabilities of the osteosarcoma cells *in vitro*

**Figure 4:**
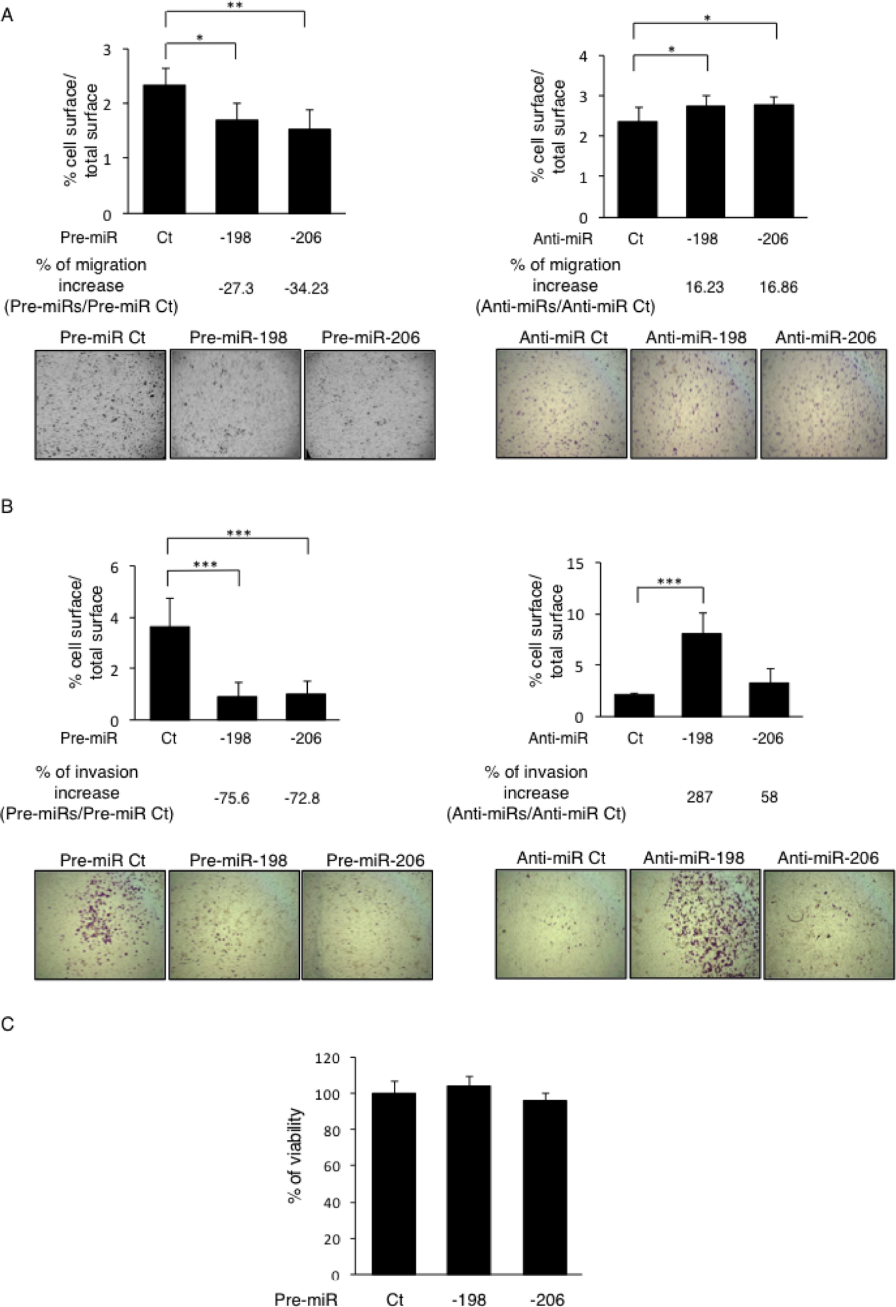
miR-198 and -206 inhibit both the migrative and invasive capabilities of the osteosarcoma cells *in vitro* (**A**) The migrative capabilities of the Osteosarcoma cells were assessed by migration assays in Boyden Chambers. The HOS LucF-GFP cells were transfected with the indicated pre-miRs (left panels) or anti-miRs (right panels) and 30 000 cells were seeded on the upper surface of the 8 µm-pored-Chambers in a 1% FBS-containing medium. The cells that had migrated to the lower surface of membranes after 24 h of incubation were counted. Histograms show the percentage of the lower membranes’ surface occupied by cells / the total lower membranes’ surface. Representative pictures were taken. Error bars show s. e. m. for *n =* 3 measurements from representative experiments. (**B**) The invasive capabilities of the cells were assessed as the same way as presented in (A), the upper surfaces of the Boyden Chambers were preliminary coated with 50 ng of Matrigel. (**C**) The viability of the cells was assessed through WST-1 assays in HOS LucF-GFP cells 72 h after the cells’ transfections with the pre-miRNAs -198, -206 or controls (Ct). ^*^p < 0.05, ^**^p < 0.01, ^***^p < 0.001. Error bars show s. e. m. for *n =* 3 measurements from representative experiments. One-way ANOVA followed by Dunnett’s multiple comparison test was used to assess the significance of the results in the experiments presented here.

As previously described in the literature and as sustained by our results, it is well established that the C-Met receptor is implicated in the control of several signaling pathways, including the ERK1/2 and the Akt ones, which further regulate the cellular proliferation, migration and invasion. In line with this consideration and given the C-Met’s targeting capabilities of the miR-198 and -206, the functional effects of these miRNAs on cellular migration was assessed by performing *in vitro* Boyden Chamber assays. The two miRNAs studied display significant effects on the migrative potential of the Osteosarcoma cells *in vitro*, as the pre-miR-198’s transfections decreased the migration by 27.3% whereas the pre-miR-206’s ones reduced it by 34.2% compared to the Control pre-miR’s conditions (Figure 4A, left panel). Consistently, inhibiting these endogenous miRNAs with anti-miRNAs stimulated the cells’ migration (by 16.23% and 16.86% for the anti-miR-198 and the -206 one, respectively) compared to the Control anti-miR (Figure 4A, right panel). Such results thus sustain the inhibitory effect of these miRNAs on the migrative capabilities of the Osteosarcoma cells.

For further evidence, we also performed a wound-healing assay in which a scratch was done in the HOS cells’ layer, 48h after their transfection with the candidate pre-miRNAs or anti-miRNAs. In such assays, the pre-miRNAs-transfected cells display early reduced recovering features, still noticeable 12 hours after performing the scratch, compared with the control conditions (Supplementary Figure 1A, left panel). Withal, the stimulating effects of the anti-miRNAs on the migration were barely detectable in this assay (Supplementary Figure 1A, right panel).

We then wondered if the miR-198 and -206 also modulate the invasive properties of the Osteosarcoma cells. Matrigel-coated Boyden Chamber assays reveal that in the pre-miRNAs’ transfections’ conditions, the invading cells’ proportion was decreased by more than 70% for each miRNA, compared to the control conditions (Figure 4B, left panel). Moreover, the transfections with the antagonists, especially the one with the anti-miR-198, induced an increase in the invasive capacities of the cells, which were enhanced by 287% in this case (Figure 4B, right panel).

As the invasive capabilities of the cells are often correlated with the expression of the Matrix Metalloproteinases (MMPs), we wondered if the miRNAs of interest inhibit the Osteosarcoma cell’s invasiveness through modulating the expression of c-Met and indirectly the expression and/or the activity of the MMPs. Our qPCR results show that the miR-133b, -198 and -206 are all able to reduce the MMP1’s expression whereas neither seem to impact the MMP2’s one (Supplementary Figure 1B). In addition, only the miR-206 displays a slight inhibitory effect on the MMP9’s expression. We complete this study by assessing the activity of the MMPs in the culture-media through zymography assays from the proteins secreted by the malignant cells. The results show that both the MMP2’s and the MMP9’s activities are reduced after increasing the miR-133b’s expression only (Supplementary Figure 1C). Taken together, this body of data argues for the crucial role played by the miR-198 and -206, not only on the migration, but also in the invasion process, two prerequisite steps contributing to the metastatic-nodules formation.

As the cell survival is another biological aspect sustained by the activation of the C-Met receptor and its downstream pathways, we finally checked the implication of the miR-198 and -206 in modulating this component. However, the WST-1 assays results show no significant differences in terms of viability in cells overexpressing either the miR-198 or the miR-206 compared to the control cells (Figure 4C). Nonetheless, this data sustains that these miRNAs’ effects, previously observed on both the migrative and the invasive capabilities of the Osteosarcoma cells, are only attributable to their inhibitory effects on pathways directly related to these functions and are not caused by modulations in the cell survival, arguing still here for their anti-metastatic role.

### The miR-198 and -206 reduce the metastatic spreading of osteosarcoma in a HOS LucF-GFP xenograft model and prolong cancer-specific survival

**Figure 5:**
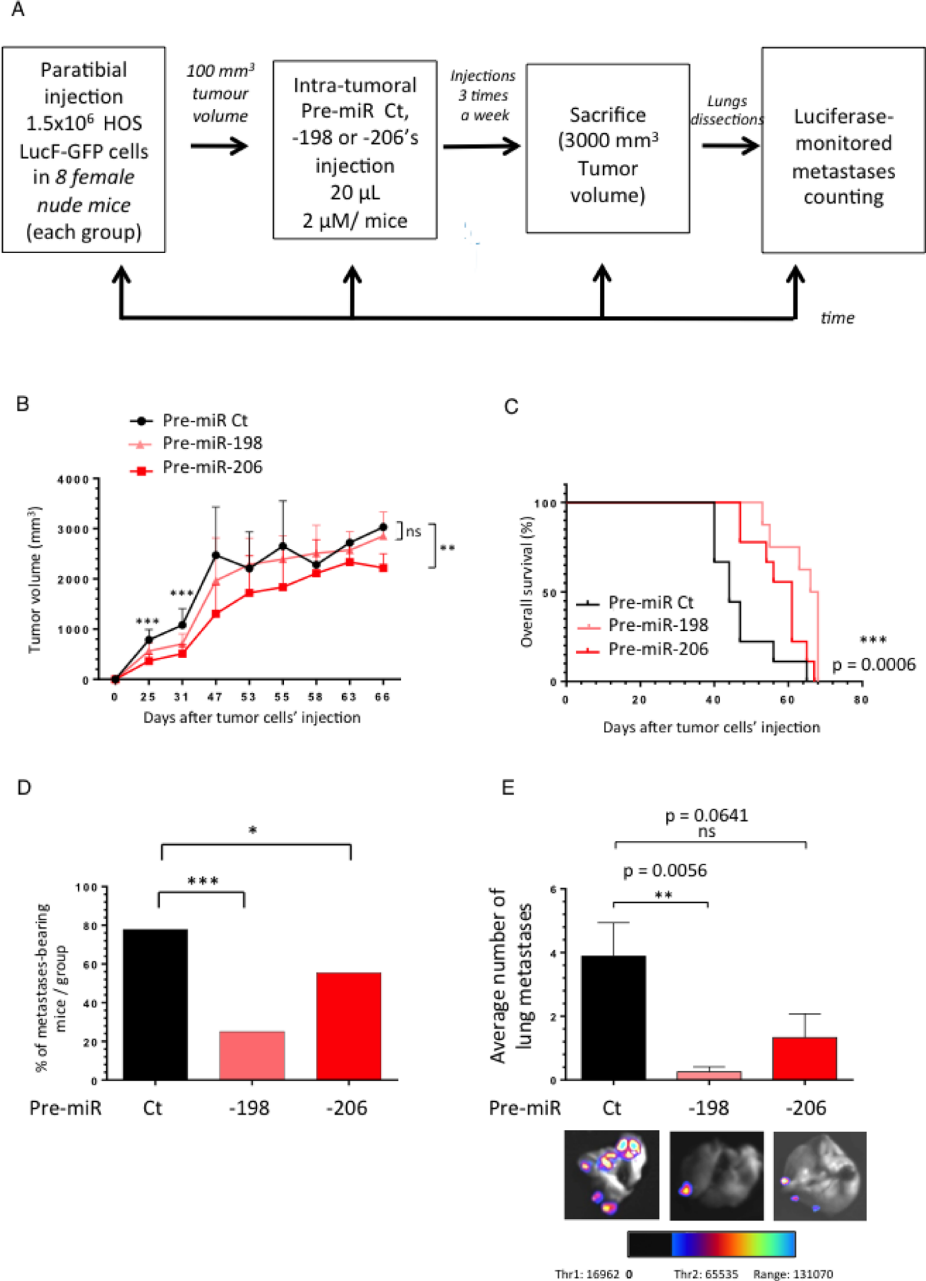
The miR-198 and -206 reduce the metastatic spreading of osteosarcoma in a HOS LucF-GFP xenograft model and prolong cancer-specific survival (**A**) Experimental design: 1.5 million HOS LucF-GFP Osteosarcoma cells were paratibially injected in nude mice. Three groups of eight mice were then randomly assigned and each one was intra-tumorally injected with either the Pre-miR Ct, the -198 or the -206 ones three times a week since the tumor volumes reached 100 mm^3^. The tumor growth was monitored until the time of the euthanasia, when the tumors were approximately 3000 mm^3^. At this time, the lungs were dissected and subjected to a luciferase-monitored counting of the metastases. (**B**) The mean tumor volume of the treated-mice was compared with control group ± s.e.m. (*n =* 8 mice in each group). A Two-way ANOVA statistical test was used to compare the effect of the different miRNAs on the tumor growth. (**C**) In Kaplan–Meier curves, cancer-specific survival were compared between mice treated with Pre-miR Ct, Pre-miR-198 and -206. The log-rank (Mantel-Cox) test was used to compare the overall survival between the three groups. (**D**) Histograms represent the percentage of the lung metastases-bearing mice depending on the treatment-groups. (**E**) Histograms show the average number of metastases counted thanks to luciferase-monitoring in each group of mice. Representative overlay illustrating pictures were presented. ^*^p < 0.05, ^**^p < 0.01, ^***^p < 0.001.

To finally support the *in vitro* anti-migrative capabilities of the miR-198 and -206, we assessed their power in further inhibiting the *in vivo* metastatic dissemination of Osteosarcoma. We thus used an Osteosarcoma xenograft model in which athymic mice were paratibially injected with 1.5 million HOS LucF-GFP cells. When the tumor volumes reached approximately 100 mm^3^, the mice received the pre-miR Control (Ct), the pre-miR-198 or the pre-miR-206 as intra-tumoral injections, three times a week (Figure 5A). The average tumor growth was followed in each group until the time of euthanasia. At that time the lungs were dissected and subjected to a luciferase-monitored counting of the metastasis number.

The miR-198 doesn’t display a significant effect on the tumor growth after the forty-seventh day following the tumor cells injection while the miR-206’s injected group shows a slowed tumor progression (Figure 5B). Furthermore, the intra-tumoral’s miRNAs’ injections significantly improve the overall mice-survival (Figure 5C).

Moreover, although about 78% of the animals develop metastasis in the Control group, only 25 and 56% display any secondary lung-metastases in the pre-miR-198 and -206 injected groups, respectively (Figure 5D). In addition, both miRNAs reduce the number of the lung-metastases developed by the animals, especially the miR-198 which decreases by about four times the average metastatic spreading compared with the Control miRNA’s injected group (Figure 5E). In parallel, we observed that the reduced expression of both the miR-198 and the miR-206 in the metastases samples compared with the primary tumors ones is less pronounced in the pre-miR-206’s injected mice group than in the Control one (Supplementary Figure 2A, 2B). In conclusion, the injection of these miRNAs not only impacts the number of the animal-bearing metastasis, but also the average metastasis number per animal.

In addition, the protein expression of C-Met in PT samples from the miR-206’s injected group is reduced compared with the Control one (Supplementary Figure 2C, 2D), thus reinforcing the demonstration of the *in vivo* inhibitory effect of this miRNA on its target and its consequent impact on the metastasis occurrence.

These data bring the proof of concept of the use of these miRNAs as potential therapeutic tools in order to control the metastatic spreading of the Osteosarcoma.

## DISCUSSION

The metastatic spreading of the tumor’s cells is one of the main causes of the cancer-therapies’ failure, especially in Osteosarcoma in which only few therapeutic options are available. As this multistep-process depends on the intrinsic properties of the tumor cells and the treatment-responses from the patients, it seems by far obvious that this evasion-mechanism is regulated by both genetic and epigenetic components [26]. Its genetic-related fraction can at least partially correspond to the accumulation of oncogenes’ activating-mutations by the most genetically unstable cells within the heterogeneous primary tumor. Such mutations-bearing cells can acquire new properties, allowing them to detach from the bone-primary site, enter into the circulation, extravasate and thereby grow at secondary organs, preferentially the lungs in the case of the Osteosarcoma [27].

Furthermore, beyond the mutational processes undergone by oncogenes, their epigenetic modulation through the miRNA-silencing machinery is another alternative way to control their expression level, consequently impacting the metastatic dissemination. Even if some studies have already demonstrated the implication of such tumor-suppressor miRNAs in the metastatic-dissemination of Osteosarcoma, their precise functions still need to be deciphered in this context. For instance, it was reported that the miR-26b inhibits the metastatic spreading of this cancer through both its CTGF and Smad1 direct targeting [28]. In addition, the miR-33b-mediated inhibition of C-Myc was reported to decrease both the migrative and the invasive properties of the MG63 Osteosarcoma cells *in vitro* [29]. Therefore, identifying new miRNAs/targets couples involved in the metastatic progression of Osteosarcoma appears of paramount interest in an attempt to use them as biomarkers to improve the diagnostic, the prognostic and the outcome of this rare malignancy.

Here, we report that human primary Osteosarcoma samples from *in vivo* xenograft models harbor a different miRNA-signature compared with their associated distant lung-metastases. We found that among the most differentially expressed miRNAs, four in particular, namely; the miR-133b, -198, -206 and -582-5p are only expressed in the cells from bone-primary sites (Figures 1C, 1D, 2A). Interestingly, the loss of expression of these miRNAs was confirmed in metastatic samples from Osteosarcoma patients compared with their primary tumor biopsies (Figure 2B).

Over both *in vitro* and *in vivo* experiments, we have more precisely defined that the down-deregulation of the miR-198 and -206 are associated features allowing the metastatic spreading of the Osteosarcoma cells. Indeed, by modulating the expression of these miRNAs *in vitro*, we highlighted their functional implication in regulating both the migrative and the invasive capacities of the cells (Figure 4A, 4B and Supplementary Figure 1A). In addition, we confirmed that such functional effects were at least partially mediated through these miRNAs’ direct binding-capabilities towards the 3′UTR of the tyrosine-kinase receptor C-Met, as previously described in other models (Figure 3D) [24, 25]. Furthermore, our results are in accordance with another study, reporting that inhibiting the expression of this receptor through a siRNA-mediated strategy or by using a pharmacologic inhibitor, the Crizotinib, prevented the uveal melanoma’s metastatic spreading [30]. In addition, it was shown that the expression of this receptor was gradually increased during the course of the colorectal cancer development, as well as it was correlated with its liver-metastatic dissemination [9]. In line with this data, we have also shown that the C-Met’s expression was up-regulated in the lung-metastases from our mice Osteosarcoma xenograft model and, even more importantly, in the ones from Osteosarcoma’s patients (Figure 2A, 2B).

As the metastatic dissemination-related functional effects of the miR-198 and -206 are at least partially attributable to their direct targeting of C-Met (Figure 3A, 3B), we have also demonstrated that they consequently act on the activation levels of two of its downstream pathways, the ERK ½ and the Akt ones (Figure 3D) [31, 32]. Besides, a recent work highlighted the implication of the ERK ½ signaling in the metastatic dissemination of the Osteosarcoma cells, as it reports that a (-)-epigallocatechin-3-gallate treatment markedly inhibits both the migrative and the invasive capabilities of the cells, as well as it reduces the P-ERK level [33]. Additionally, it was also demonstrated that the EMT is mediated by the ERK ½ pathway in Osteosarcoma cells [34]. Moreover, the crucial role of the PI3K/Akt pathway in the metastatic development of Osteosarcoma was also reported, this pathway displaying a significant higher activation level in lung-metastasis bearing patients compared with the non-metastatic ones [35]. Interestingly, the PI3K/Akt pathway is also linked to the EMT and the invasive capabilities of the Osteosarcoma cells, as it contributes to the Matrix Metalloproteinases’ activation. It was indeed reported that inhibiting this pathway decreases both the MMP2 and the MMP9 activities, further impeding the metastatic spreading of the murine Osteosarcoma cell line LM8, nevertheless highly aggressive [36]. This data are linked to the anti-invasive potential of the miR-198 and -206, here rather associated with their inhibitory effect on the MMP1’s expression (Figure 4B and Supplementary Figure 1B).

Furthermore, in a therapeutic approach, this study brings the proof of concept that the miR-198 and -206 could be employed as powerful treatment-tools in the Osteosarcoma context. To our knowledge, it is indeed the first time that the direct intra-tumoral injection of these miRNAs was performed in a Bone Sarcoma model. Our study shows that, used as therapeutics, these miRNAs reduce both the tumor growth and the metastatic-spreading, further resulting in increasing the mice’s overall survival (Figure 5). Besides, our data even strengthen the relevance of using these miRNAs in an Osteosarcoma’s context, as the anti-migrative and the anti-invasive roles of the miR-198 were previously demonstrated in this pathology, due to its direct binding to another target gene : ROCK1 [37]. In addition, these authors demonstrated that the expression level of this miRNA was correlated with the TNM stage and the metastatic spreading of the disease. In gastric, colorectal and lung cancers a decrease in the miR-198’s expression was also correlated with the metastasis occurrence [38–40]. Furthermore, Osteosarcoma patients displaying low levels of miR-206 and -133b in tumor and sera were shown to exhibit high Osteosarcoma grade as well as distant metastasis [41, 42].

Such results complete the mode-of-action-related knowledge about the miR-198 and -206 and shed light on their crucial role in the metastatic spreading of the Osteosarcoma, at least through their direct C-Met targeting. In a clinical context, this work thus aims to open the road to new therapeutic opportunities provided by the use of a synthetic version of these molecules, in an attempt to improve the outcome of this pediatric cancer.

## MATERIALS AND METHODS

### Tumor cell lines and patient tumor material

The HOS LucF-GFP cells are modified MNNG/HOS cells (young female high grade Osteosarcoma from femoral origin, transformed *in vitro* by N-methyl-N’-nitro-N-nitrosoguanidine treatment) that include the Firefly luciferase (LucF) and the Green Fluorescent protein (GFP) genes stably inserted in their genome. Cell modification was done by lentiviral infection following the procedure previously described to modify mouse POS-1 and rat OSRGA cell lines [43]. The cells were cultured in Dulbecco’s modified Eagle’s medium (DMEM; Invitrogen-Life Technologies Inc.) supplemented with 10% fetal bovine serum (FBS; Hyclone), 2 mmol/L L-glutamine and 1% penicillin/streptomycin. The cells were maintained in a humidified 5% CO_2_/air atmosphere at 37° C and were passaged for less than 3 months.

Osteosarcoma cell lines sub-populations were obtained at the time of diagnostic biopsy (B) or after surgical resection of lung metastasis (M), in patients diagnosed with Osteosarcoma at the Hospital of the University of Navarra (Clínica Universidad de Navarra, CUN, Pamplona, Spain). Samples were obtained following patient informed consent and after ethical approval by the Navarra University Hospital Ethics Committee. All sub-populations were thoroughly characterized as previously described by Patiño-García *et al.*, [44]. The Supplementary Table 1 contains all the patients’ details.

### Animal treatment

All procedures involving mice (their housing in the Experimental Therapeutic Unit at the Faculty of Medicine of Nantes (France) and care, the method by which they were anesthetized and killed and all experimental protocols) were conducted in accordance with the institutional guidelines of the French Ethical Committee (CEEA.PdL.06). MNNG/HOS osteolytic xenograft model was induced by paratibial injection of 1.5 × 10^6^ of HOS LucF-GFP cells in five weeks-old female athymic nude mice (Harlan Sprague-Dawley, Inc.), leading to a rapidly growing tumor in soft tissue with secondary contiguous bone invasion. The tumor growth was monitored three times weekly and tumor volumes were calculated by using the formula: length^*^width^*^depth^*^0.5. Data points were expressed as average tumor volume ± s.e.m. until a maximum of 2500 mm^3^.

Mice were anesthetized by inhalation of a combination of isoflurane/air (1.5%, 1L/min) and blood was collected in tubes containing EDTA (1.5 mg ± 0.35 mg EDTA/mL of blood) by intracardiac puncture. Blood samples were incubated with a red-cells lysis buffer (composition: 8.26 g NH4Cl, 1 g KHCO3, 200 μL EDTA 0.5 M, pH 8; all together diluted in 100 mL ddH_2_O; filtered and adjusted to pH 7.4) and the remaining cells were counted and diluted in PBS 2% FBS + 0.7 mM EDTA. From *in vitro* culture 1 × 10^6^ HOS LucF-GFP cells were trypsinized and diluted in PBS 2% FBS + 0.7 mM EDTA and served as a control for CTCs’ isolation. Cell sorting was made in the cell sorting facility SFR/INSERM U892. Primary tumor samples and lungs from each animal were also collected. Lungs observation and further metastasis dissection and collection were done under an optic microscope. RNA was extracted from all samples.

### Luciferase-monitored counting of the metastasis

The mice were sacrificed seven minutes after they received an intra-peritoneal injection of 250 µL of a luciferine solution. The lungs were dissected and the EG&G Berthold Night Owl *In-vivo* Molecular Imaging System facility associated to the WinLight 32 software, were used to monitor the disseminated HOS LucF-GFP cells and to count the lung-metastasis.

### RNA extraction

Cultured cells and *in vivo* samples were lysed with 700 μL of “QIAzol^®^ Lysis Reagent” (QIAGEN, CA, USA). Messenger RNA (mRNA) and miRNA were extracted with the “QIAGEN miRNeasy Mini Kit” (QIAGEN) following manufacturer’s instructions. The mRNA concentration was measured by optical density (OD) at 230, 260 and 280 nm thanks to a spectrophotometer (Nanodrop, Thermo Scientific).

### Taqman low density array (TLDA)

RNAs from the isolated CTCs, PTs and MET samples were sent to the IGGM *(Institut de Génétique Moléculaire de Montpellier)* where the expression of 760 miRNAs was analyzed by Taqman Low Density Array (TLDA). To obtain the miRNAs’ expression profiles of the above-mentions samples, data was normalized with three reference miRNAs (RNU6B, RNU44 and RNU48), with respect to primary tumors and a 2-fold difference was considered as significant threshold regarding each miRNA’s expression variation. The results from two independent experiments were compared.

### Quantitative reverse transcription–PCR

The mRNA was retro-transcribed into complementary DNA (cDNA) with the ThermoScript™ RT-qPCR System (Invitrogen), following the manufacturer’s instructions. Real-time monitoring of PCR amplification of cDNA was performed using DNA primers (primers sequences are available in Supplementary Table 2) on CFX96 real-time PCR detector system (Bio-Rad, Marnes la Coquette, France) with SYBR PCR Master Mix buffer (Bio-Rad). Target genes expression were normalized to glyceraldehyde 3-phosphate dehydrogenase (GAPDH) and β-2 microglobulin (B2M) levels in respective samples as an internal standard and the comparative cycle threshold (Ct) method was used to establish a relative quantification of target mRNAs. Each assay was performed in triplicate.

A specific reverse-transcription (RT) was performed for each miRNA from 100 ng of total RNA, using a specific stem-loop RT primer (50 nM) and the MultiScribe Reverse transcriptase (Applied Biosystems). The mature miRNAs’ expression levels were measured by Real-time qPCR using the SYBR PCR Master Mix buffer (Bio-Rad) and a CFX96 real-time PCR detector system (Bio-Rad). The expression of each gene was normalized to the one of the small nuclear RNU6B RNA as a reference. All experiments were performed in triplicate. Primers sequences are available in Supplementary Tables 3 and 4.

### Transfections

MirVana™ miRNA mimics (pre-miRNAs) or mirVana™ miRNA inhibitors (anti-miRs) (Ambion, Invitrogen) were transfected at 30 nM final concentration thanks to the siPORT™ NeoFX™ Transfection Agent (Invitrogen), following manufacturer’s instructions.

### Western blotting analysis

Samples containing equal amounts of protein (depending on the antibody, 5–50 µg) from lysates of cultured Osteosarcoma cells underwent electrophoresis on SDS-polyacrylamide gels and were transferred to polyvinylidenedifluoride (PVDF) membranes. The membranes were blocked in 3% BSA-PBS-0.05% Tween at room temperature for 1 h and blots were probed overnight at 4° C with the following primary antibodies : rabbit anti-MET (C-12), 1:500; Santa Cruz Biotechnology, rabbit anti-Akt #9272S, 1:1000; rabbit anti-P-Akt (S473) #9272S, 1:1000; rabbit anti-p44/42 #9102S, 1:1000; rabbit anti-P-44/42 MAPK (T202/Y204) #4370S, 1:2000; or rabbit anti-GAPDH 14c10, 1:2000; Cell Signaling Technologies, Beverly, CA, to detect proteins of interests. After incubation, the membranes were washed 3 times with washing buffer (PBS containing 0.05% Tween) for 5 min. Membranes were then incubated for 1 h with 1:10,000 diluted secondary antibody (goat-anti-rabbit sc-2004 #J1512, 1:10000; Santa Cruz Biotechnologies, Santa Cruz, CA) at room temperature. Specific proteins were detected using SuperSignal^®^ WestDura Extended Duration Substrate (ThermoScientific, Rockford, USA) and a G-Box (Syngene, Cambridge, UK) after washing. Pictures were analysed thanks to the ImageJ software. Glyceraldehyde-3-phosphate dehydrogenase was used as an internal loading control.

### Luciferase reporter assay

HOS LucF-GFP cells were cultured in 24 well-plate (40,000 cells/well) and transfected with 10 ng of either control reporter vector (Psi-ckeck2 control) or UTR-reporter vector (Psi-check2 C-Met 3′UTR) together with pre/anti-miRs control, -133b, -198 and -206 (15 μM) following manufacturer’s recommendation thanks to the siPORT™ NeoFX™ Transfection Agent (Invitrogen). Forty-eight hours after transfection, cells were lysed and the luciferase activity was measured with the “Dual-Luciferase^®^ Reporter Assay System” kit (Promega). 25 μL of substrates for Renilla and Firefly luciferases are added each time to the lysed cells and the resultant bioluminescence was measured with TriStar LB 941 (Berthold Technologies). Psi-check2 C-Met 3′UTR was a kind gift from Dr. Chonglin Luo German (Cancer Research Center (DKFZ), Heidelberg, Germany) in which the C-Met 3′UTR sequence was inserted between the NotI and XhoI sites [45].

### Migration and invasion assays

HOS LucF-GFP cells were transfected with the indicated pre-miRs or anti-miRs and 30 000 cells were seeded on the upper side of a Transwell Chamber (Falcon), on a porous transparent polyethyleneterephthalate (PET) membrane (8-μm pore size) in 1% FBS. The lower chamber of the Transwell was filled with growth medium containing 10% FBS. Such 1%/10% FBS-gradient was generated between the upper and the lower Chambers of the system, to promote the cell migration. After 24 hours of incubation, cells on the upper side of the Chambers were mechanically removed and cells that migrated to the lower side were fixed with 10% Glutaraldehyde and stained with 0.1% Crystal Violet. Pictures of the Chambers were taken and five different areas were arbitrary chosen to perform quantitative analyses. Representative pictures of the Boyden were chosen here. For all the Boyden Chambers experiments, error bars show the standard deviation for *n =* 8 measurements from representative experiments and two-tailed paired Student’s *t-tests* were used to compare the different conditions.

The same procedure was used to monitor the invasive capabilities of the cells, with this difference that the upper side of the Transwell Chambers were Matrigel-coated (50 ng Matrigel/well).

### Time-lapse scratch assays

HOS LucF-GFP cells were plated in 24-well plate and transfected with the indicated pre/anti-miRs (50 nM). 48 hours later and upon cell confluence, scratches on the center of the wells were performed thanks to pipet-tips. The scratch recovery was recorded for 24 h and pictures were taken every 10 minutes. Calculations were made with AxionVisionRel 4.8 software (Zeiss).

### Viability assays

HOS LucF-GFP Osteosarcoma cells were plated in 96-wells plates (2000 cells/well) and transfected with the pre-miRs, as previously mentioned. The cell viability was evaluated with WST-1 solution (4-[3-(4-Iodophenyl)-2-(4-nitrophenyl)-2H-5-tetrazolio]-1,3-benzene disulfonate, Roche, Mannheim, Germany). 72 hours after the transfections, the culture medium was removed and replaced by the WST-1 reagent diluted in fresh medium in a 1:10 proportion. After a 7 hours incubation time, the absorbance at 470 nm was measured for each well on a 96-multiwell microplate reader (Victor^2^ 1420; PerkinElmer Inc.) and normalized to the average reading of wells containing medium only. Each assay was performed in triplicate. The viability percentage is calculated by this formula: OD at 470 nm with indicated pre-miR/OD at 470 nm with Control pre-miR × 100.

### Statistical analysis

All error bars show SEM (s.e.m.) or SD (s.d.) for at least triplicate measurement from representative experiments. Statistical tests were done with GraphPad Prism 6 software (^*^=p ≤ 0.05, ^**^=p ≤ 0.01, ^***^=p ≤ 0.001). In the case of comparing two samples, two-tailed Student *t*-tests were performed, whereas multiple comparisons were analysed by one-way ANOVA tests followed by a Dunnett’s test. The test used in each case is indicated on the legend of the corresponding figure. Error bars from the *in vivo* tumor growth monitoring represent the s.e.m. from the mean tumor volume of the mice (*n =* 8 mice in each group). A *p* < 0.05 was used as the criteria for statistical significance.

## SUPPLEMENTARY MATERIALS FIGURES AND TABLES


