# Supplementary material for: Loss of miR-198 and -206 during primary tumor progression enables metastatic dissemination in human osteosarcoma
Source: Oncotarget. 2018 Nov 6;9(87):35726–41. doi: 10.18632/oncotarget.26284 (PMC6254661; doi:10.18632/oncotarget.26284)
Supplement: Supplementary file 1 [file oncotarget-09-35726-s001.pdf]

Loss of miR-198 and -206 during primary tumor progression enables metastatic dissemination in human osteosarcoma

SUPPLEMENTARY MATERIALS

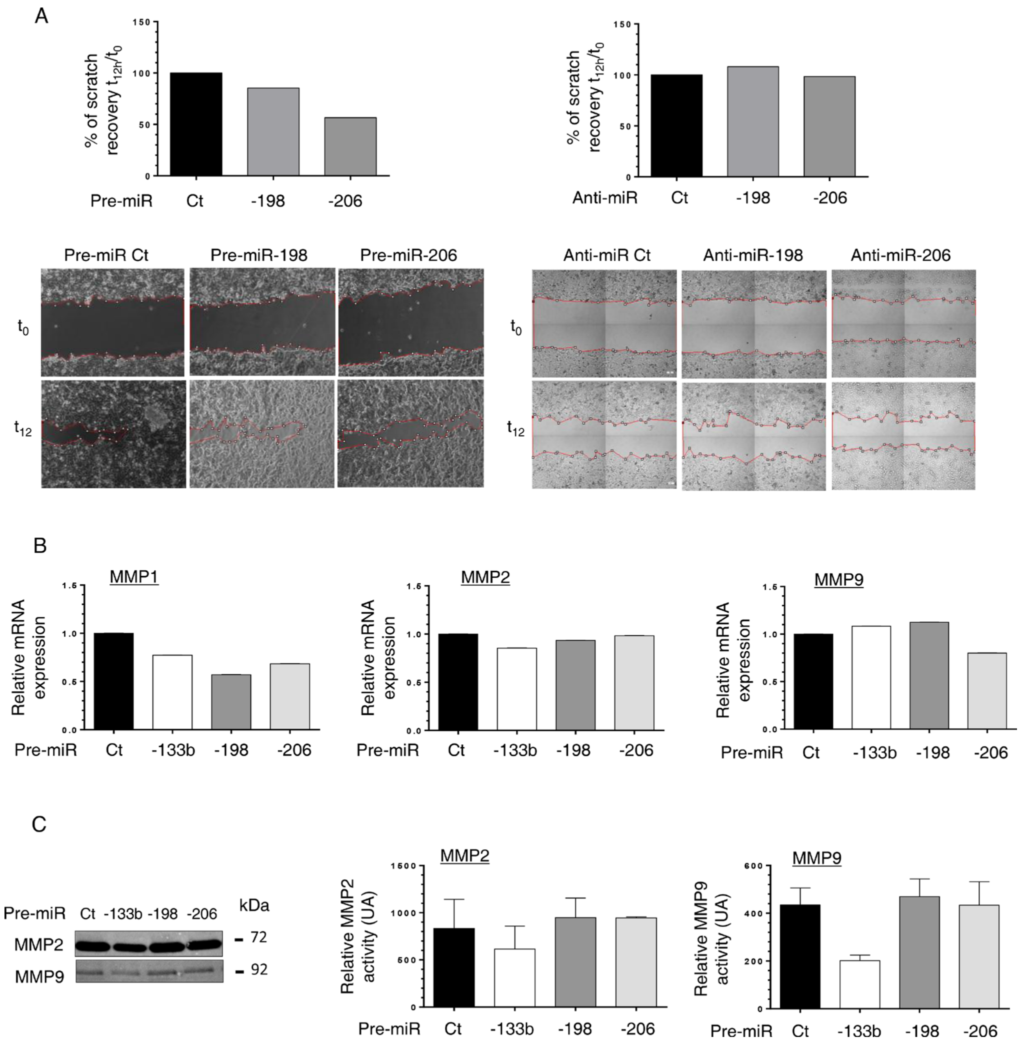

Supplementary Figure 1: The miR-198 and -206 inhibit the *in vitro* migrative and invasive capabilities of the osteosarcoma cells through impacting both the matrix metalloproteinases expression and activity. (**A**) The HOS LucF-GFP cells were transfected with the indicated pre-miRNAs (left panel) or anti-miRNAs (right panel) and a wound was generated forty-eight hours later on the cell layer, after cell confluence. Histograms show the recovery percentages in each transfection's condition 12 hours after performing scratches, with respect to the corresponding condition at t_0. Pictures were taken at the initial time-point (t_0) (upper panel) and 12 hours after performing the scratches (t_{12}) (lower panel). The scratch areas were outlined in red. (**B**) The expression of MMP1, MMP2 and MMP9 was assessed by RT-qPCR in HOS LucF-GFP Osteosarcoma cells, 72 hours after transfections with the indicated Pre-miRNAs. Error bars show s.d for $n = 3$ measurements from representative experiments and GAPDH was used as housekeeping gene. (**C**) The HOS LucF-GFP Osteosarcoma cells were transiently transfected with the indicated Pre-miRNAs at a final concentration of 100 nM per well in DMEM-10% FBS-containing medium. Forty-eight hours later, the cells were subjected to both a starvation without FBS and another round of Pre-miRNAs' transfection at a final concentration of 30 nM per well. The supernatants were harvested forty-eight hours latter, the proteins were extracted and the Matrix Metalloproteinases' activities of the MMP2 and the MMP9 were assessed by zymography. Picture of a representative gel from two independent experiments is presented (left panel) and a relative quantization of the MMP2 and the MMP9 activity was performed thanks to the ImageJ software (middle and right panels respectively). Error bars show s.d for $n = 2$ measurements from representative experiments.

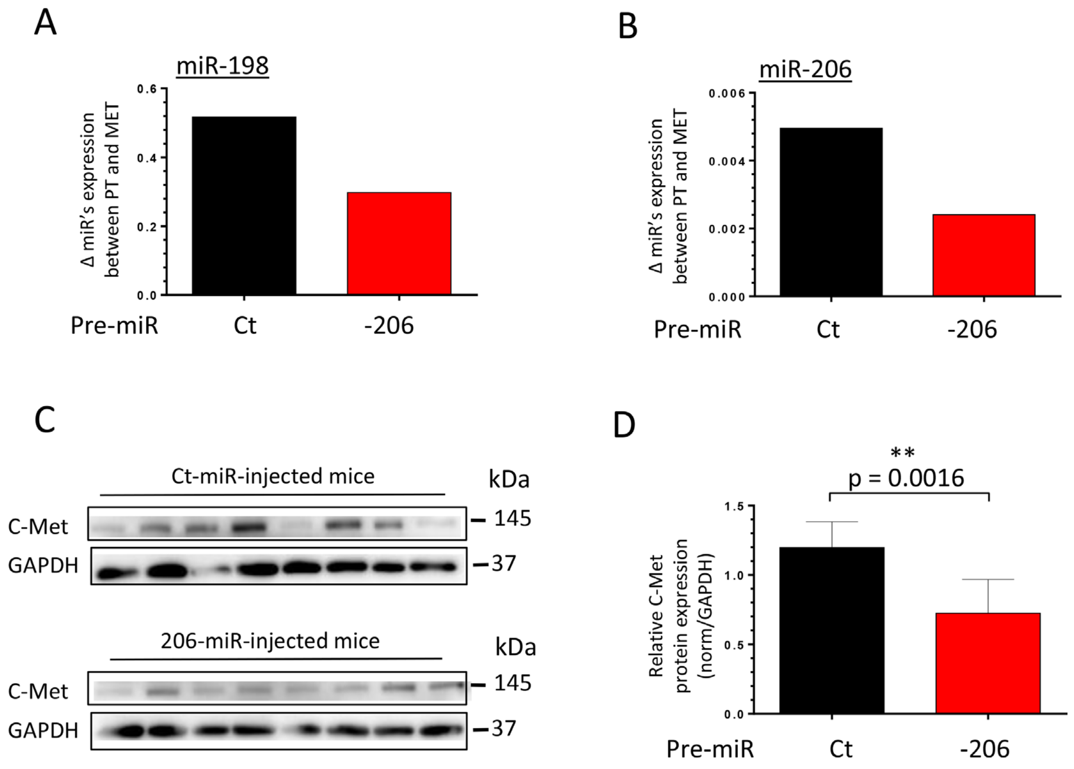

Supplementary Figure 2: Treating the mice with the miR-206 counteracts the decreased expression of both the miR-198 and the miR-206 in the metastasis. (**A**) The expressions of the miR-198 and the miR-206 (**B**) were assessed in Primary Tumors (PT) and in metastasis (MET) samples from the Control mice and the pre-miR-206's injected one by RT-qPCR. RNU6B was used as housekeeping gene. Histograms show the ratio between the miR's expression in the MET normalized on the one found in the PT for each group. (**C**) The expression of C-Met was assessed by Western Blotting in the PT from the corresponding-miR-injected mice. Each band represent the expression of C-Met in the PT from one mouse (n = 8). GAPDH was used as a loading control. (**D**) A quantization of the bands obtained in (C) was performed thanks to the ImageJ software. The average expression of C-Met was calculated for n = 7 mice per group and normalized on the expression of the GAPDH.

Supplementary Table 1: Patient's samples features and clinical data. **All the patients were diagnosed at the Hospital of the University of Navarra (Clínica Universidad de Navarra, CUN, Pamplona, Spain)**

Case	Location	Sex	Age at the diagnosis	Metastasis	Overall survival* (months)	Time to progression** (months)	Status	Primary Tumor biopsy	Metastasis biopsy
491	Femur	Male	16	YES	84	27	Dead	491Bp8	491Rp4
531	Femur	Female	22	YES	28	0	Dead	531Bp	531MIIp52
588	Tibia	Male	16	YES	46	27	Dead	588Bp10	588Mp12
595	Femur	Female	16	YES	12	0	Dead	595Bp3	595Mp11

*from diagnosis to May 2013.

**time between the end of treatment of the primary and metastasis or relapse.

Supplementary Table 2: Sequences of the forward and reverse primers used in the qPCR

Gene (human)	Forward primer (5′-3′)	Reverse primer (5′-3′)
C-Met	TCTGCCTGCAATCTACAAGG	ATTATTCCTCCGAAATCCAAAGT
B2M	AGCTGTGCTCGCGCTACTCTC	CACACGGCAGGCATACTCATC
GAPDH	TGGGTGTGAACCATGAGAAGTATG	GGTGCAGGAGGCATTGCT

Supplementary Table 3: Sequences of the Hairpin RT primers used for the miRNA-RT

miRNA	Hairpin RT primer (5′-3′)
hsa-miR-133b	GTCGTATCCAGTGCAGGGTCCGAGGTATTCGCACTGGATACGACTAGCTGG
hsa-miR-198	GTCGTATCCAGTGCAGGGTCCGAGGTATTCGCACTGGATACGACGAACCTAT
hsa-miR-206	GTCGTATCCAGTGCAGGGTCCGAGGTATTCGCACTGGATACGACCCACACAC
hsa-miR-582-5p	GTCGTATCCAGTGCAGGGTCCGAGGTATTCGCACTGGATACGACAGTAACTG

Supplementary Table 4: Sequences of the different forward and universal reverse primers used in the qPCR for the miRNAs

miRNA	Forward primer (5′-3′)	Universal Reverse primer (5′-3′)
hsa-miR-133b	CATCCTTTGGTCCCCTTCAA	GTGCAGGGTCCGAGGT
hsa-miR-198	GACAGAGGTCCAGAGGGGAG	GTGCAGGGTCCGAGGT
hsa-miR-206	GCCATCCTGGAATGTAAGGAA	GTGCAGGGTCCGAGGT
hsa-miR-582-5p	GCCATCCTTACAGTTGTTCAAC	GTGCAGGGTCCGAGGT
